# Persistent Listeria monocytogenes Cerebritis Mimicking Cerebral Metastases Due to Melanoma

**DOI:** 10.7759/cureus.62634

**Published:** 2024-06-18

**Authors:** Samantha Levin, Jordan N Houser, Hiba Siblini, Michael Marshall, Jess Wolf

**Affiliations:** 1 Neurology, Rush University Medical Center, Chicago, USA; 2 Pathology, Rush University Medical Center, Chicago, USA

**Keywords:** immunocompromise, brain biopsy, cns infection, brain metastasis, glucocorticoids, infectious disease pathology, infectious disease, malignant melanoma metastasis, new-onset seizure, listeria

## Abstract

Listeria cerebritis is a rare yet serious central nervous system infection, which can present with leptomeningeal enhancement, abscess, and seizures. An adult patient with a history of metastatic melanoma presented with left-sided weakness, later identified as postictal Todd’s paralysis due to focal motor seizures. Further diagnostic workup revealed a leptomeningeal abscess in the setting of listeria cerebritis. The patient’s condition improved after treatment with a prolonged course of ampicillin, gentamicin, and linezolid over eight weeks. Leptomeningeal disease in patients with cancer history is often thought to be metastatic disease but infections, such as listeria, should be considered even if cerebrospinal fluid is bland. Treatment of listeria may need to be prolonged in patients who are immunocompromised.

## Introduction

*Listeria monocytogenes *is a bacterial pathogen found in contaminated food of plant and animal origin, which leads to infection in adults upon consumption. The incidence of laboratory-confirmed Listeria infection in the United States is 0.24 cases per 100,000 population and causes about 260 deaths per year [1]. While listeria infection occurs as a sporadic illness of self-limited febrile gastroenteritis in healthy individuals, patients with metastatic disease and other immunosuppressive conditions are particularly at risk for listeria bacteremia and listeria cerebritis. Listeria cerebritis can present with headaches, focal neurologic signs, altered mental status, and seizures [2-4]. These same symptoms can also occur in patients with brain metastasis. Melanoma is one of the most common primary tumors associated with brain metastasis [5]. The presentation of acute-onset seizure activity in a patient with melanoma is therefore concerning for the devastating diagnosis of metastatic disease to the brain. When laboratory results and neuroimaging demonstrate concerns for metastasis, many clinicians and patients may question the value of obtaining a confirmatory diagnostic biopsy. However, other important causes of acute symptomatic seizures, such as listeria cerebritis, must also be considered [6]. This case report describes the noteworthy workup and diagnosis of listeria cerebritis in a patient with metastatic melanoma presenting with seizures.

## Case presentation

A patient in their 70s with a two-year history of melanoma with lung metastasis presented with left-sided weakness and intermittent tremors after a mechanical fall. One month before this current presentation, the patient discontinued nivolumab infusions due to drug-induced hepatitis and was treated with oral prednisone 60 mg per day, followed by a prolonged taper. The initial neurologic examination showed left upper extremity weakness and left pronator drift. Computed tomography (CT) of the head was negative for acute hemorrhage; however, a CT angiogram of the head and neck revealed a right vertebral artery dissection.

Shortly after admission, the patient had witnessed seizure-like activity and was started on levetiracetam. The left-sided weakness was then believed to be due to a postictal Todd’s paralysis. Initial limited-sequence magnetic resonance imaging (MRI) without contrast and magnetic resonance angiogram demonstrated stable vertebral artery dissection and absence of large vessel occlusion. The patient was started on aspirin. Repeat neurologic examinations showed persistent left-sided weakness, and continuous electroencephalogram monitoring was started due to concern for subclinical seizures. The patient had two more clinical seizures with maintained awareness over the next 24 hours, both originating from the right central-parietal parasagittal region. The levetiracetam dosage was increased, and lacosamide was added for additional antiseizure maintenance (ASM).

**Figure 1:**
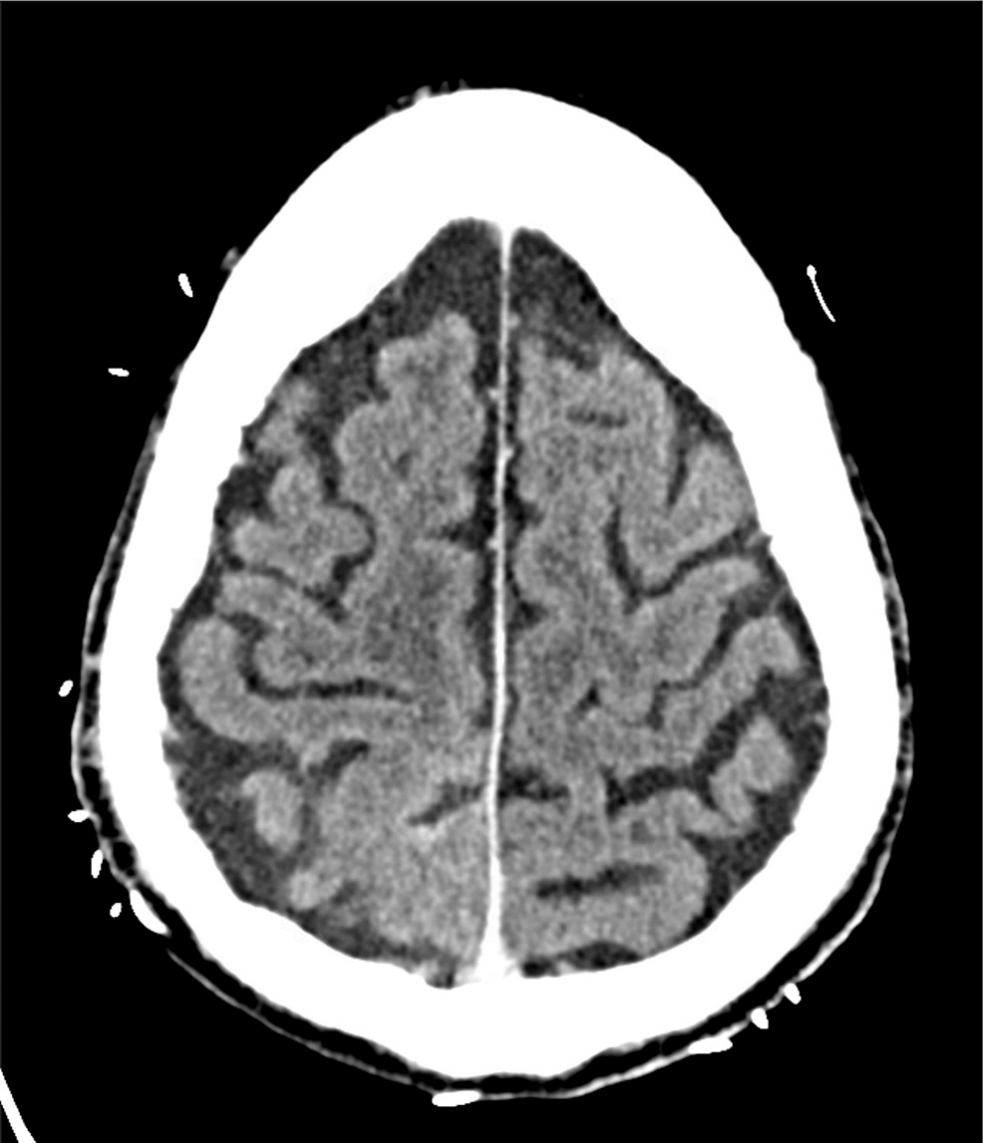
CT head with contrast; subtle contrast enhancement of right parasagittal leptomeninges CT: computed tomography

**Figure 2:**
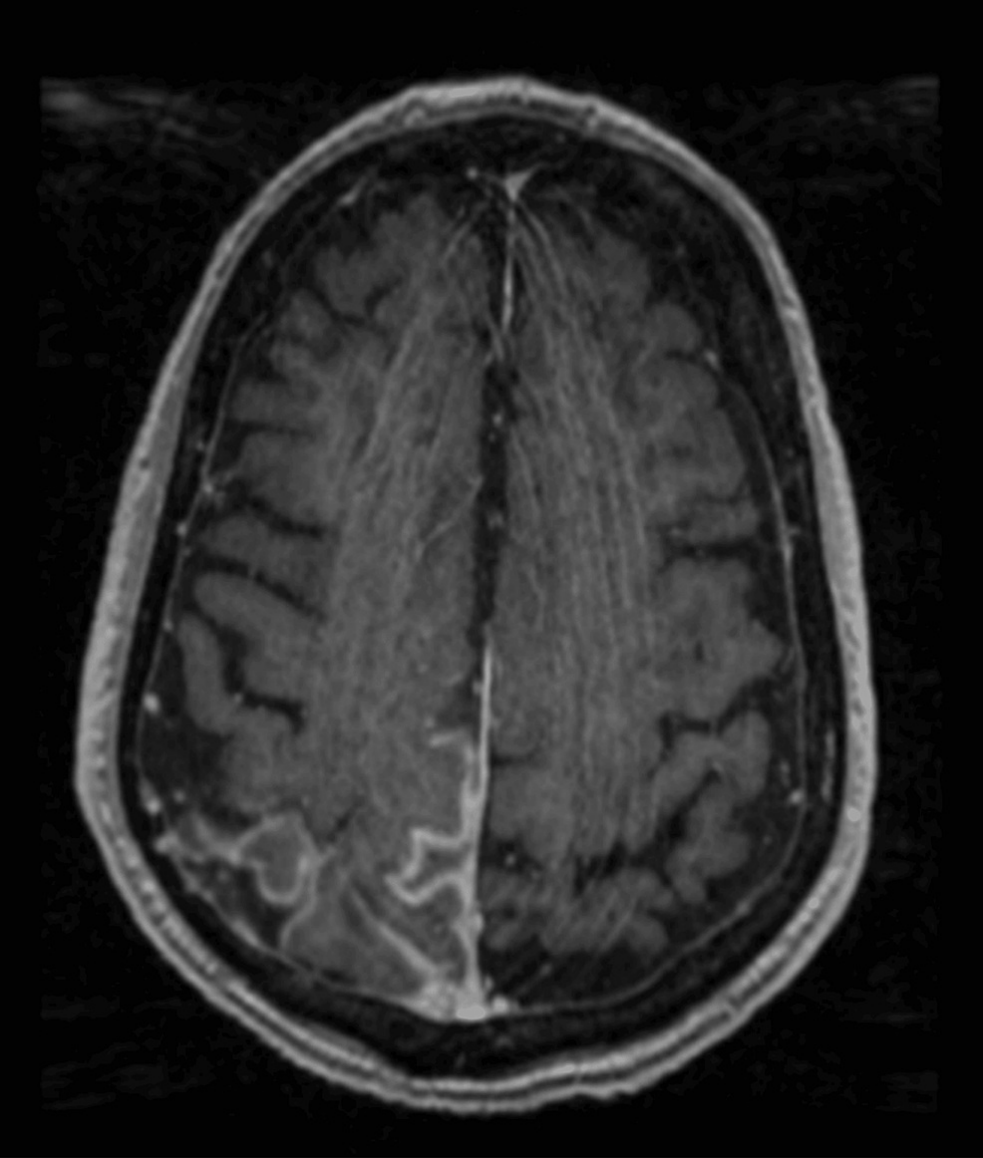
Initial MRI with contrast, fluid-attenuated inversion recovery image; leptomeningeal enhancement in the right parasagittal region MRI: magnetic resonance imaging

CT head with contrast (Figure 1) revealed a new subtle leptomeningeal contrast enhancement along the right parasagittal convexity. MRI with contrast obtained the next day (Figure 2) demonstrated leptomeningeal enhancement in the right parietal lobe with subtly associated restricted diffusion and fluid-attenuated inversion recovery hyperintensity, with no abnormal enhancement elsewhere. A differential was developed, including great concern for metastatic melanoma versus less likely infection, autoimmune encephalitis, or subacute infarct.

**Figure 3:**
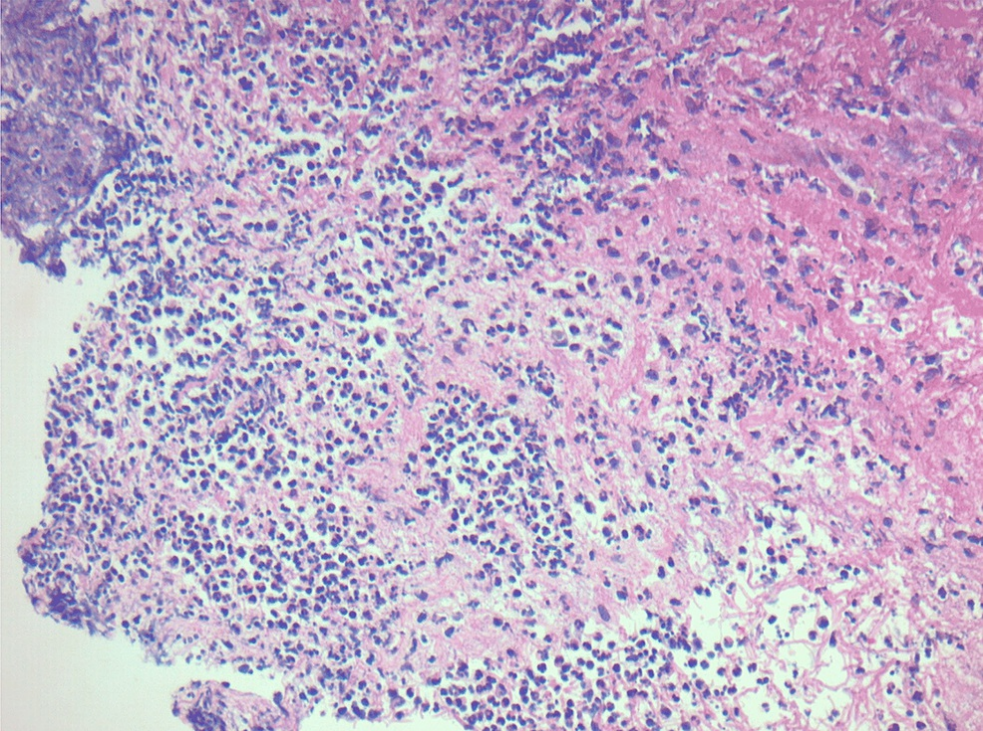
The biopsy showing sections of meninges with dense inflammatory infiltrates comprised of abundant polymorphonuclear cells with areas of necrotic debris, consistent with abscess formation

Examination of the patient’s CSF demonstrated clear fluid with a cell count of 4/µL, white blood cell count of 0/µL, red blood cell count of 2/µL, protein of 64.8 mg/dL, and glucose of 86 mg/dL with serum glucose of 126 mg/dL. CSF cultures showed no growth, and viral polymerase chain reaction testing for cytomegalovirus, herpes simplex virus 1 and 2, and varicella-zoster virus returned negative. An autoimmune encephalopathy panel of the CSF was negative as well. The patient's condition worsened, having multiple seizures per day and developing dense left-sided hemiplegia. After the patient was loaded with valproic acid, they were transferred to the Neurosciences Intensive Care Unit and intubated for airway protection while ASMs were increased. The patient developed leukocytosis and hyponatremia on hospital day 6. Given the patient’s acute decline, the decision was made to pursue a leptomeningeal biopsy, and neurosurgery was consulted. The patient went to the OR, and a biopsy was obtained without incident on hospital day 7. While awaiting the pathology results postoperatively, the patient became febrile up to 106°F. Empiric antibiotic coverage with vancomycin and piperacillin/tazobactam was started at this time. Two days later, pathology returned consistent with acute inflammation and abscess (Figure 3). *Listeria monocytogenes* was isolated from the brain biopsy cultures, and the patient was ultimately diagnosed with listeria cerebritis. Immunostaining of the tissue sample for HMB45 and SOX10, markers for metastatic melanoma, was negative.

The patient’s family reported that the patient had frequent consumption of deli meats but had no other risk factors for listeria. The patient’s immunosuppression from nivolumab treatment and subsequent prolonged steroid likely contributed.

Ampicillin and gentamicin were started, and the patient was placed on a cooling protocol, which progressively improved fevers. After appropriate antimicrobials were initiated, the patient remained seizure-free for the remainder of the hospitalization on levetiracetam and lacosamide. The neurologic exam gradually improved, and the patient was discharged on an antibiotic regimen of ampicillin and gentamicin to an acute inpatient rehabilitation facility on hospital day 25. Gentamicin was discontinued after a two-week course of treatment per infectious disease recommendation.

**Figure 4:**
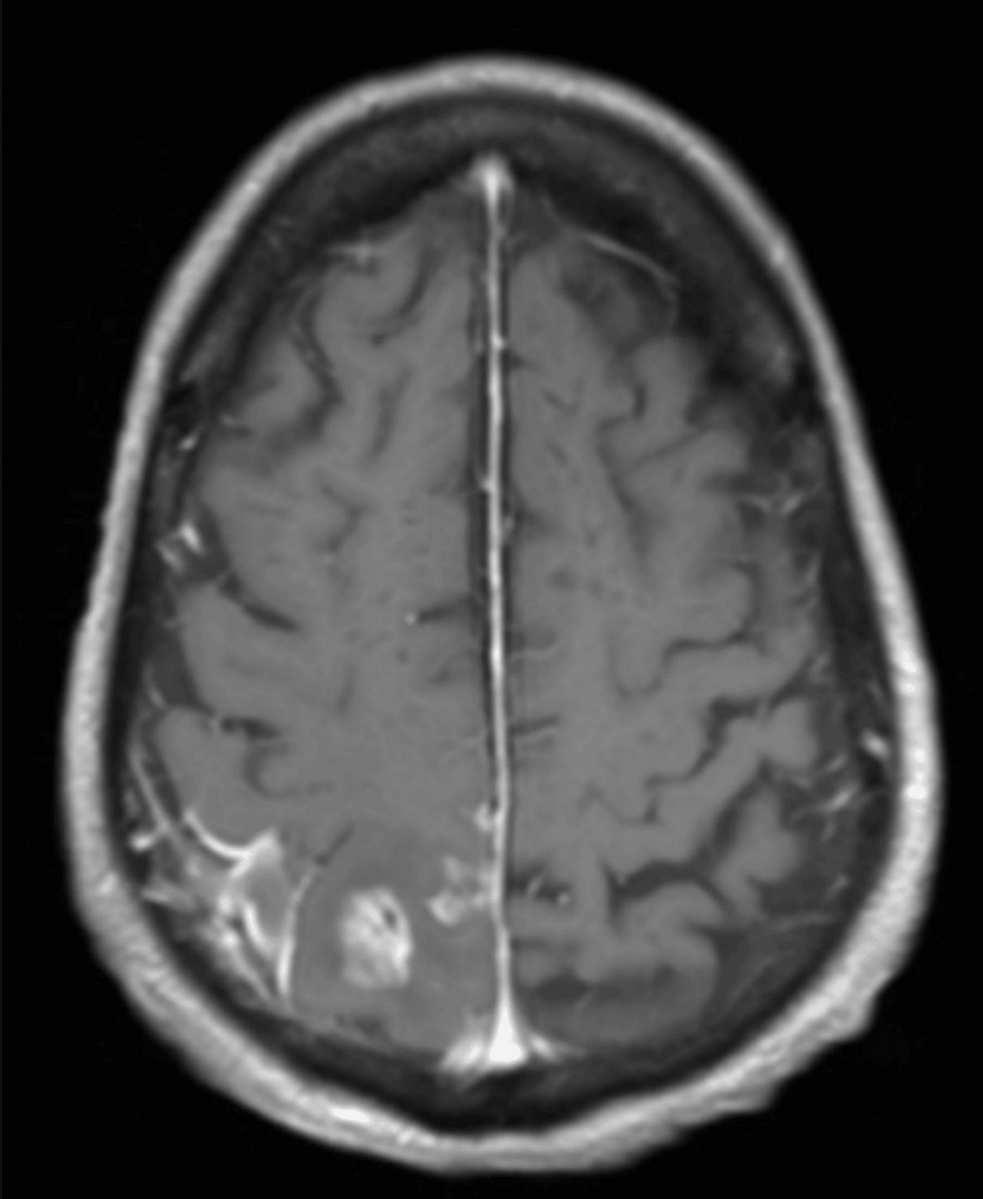
MRI with contrast, postcontrast image; worsening nodular leptomeningeal enhancement in the right parietal region with subcortical enhancing foci in the middle frontal gyrus and right postcentral gyrus MRI: magnetic resonance imaging

Unfortunately, after discharge, the patient became more somnolent. CT head imaging was repeated and showed a small hypodensity in the right parietal lobe subcortical white matter extending to the centrum semiovale. The patient was transferred back to our hospital, where they underwent an MRI with contrast (Figure 4). Imaging demonstrated worsening nodular leptomeningeal enhancement in the right frontoparietal region with areas of restricted diffusion as well as new small cortical and subcortical enhancing foci in the middle frontal gyrus and right postcentral gyrus. Lumbar puncture was performed, and CSF analysis returned with pleocytosis and elevated protein, yet no growth was detected on cultures. Given that the patient’s surgical wound remained well-appearing, there was presumed to be a larger burden of listeria infection than previously suspected, and gentamicin was resumed.

The patient’s neurologic examination continued to decline with agitation, fluctuating left-sided weakness, and poor oral intake. Out of concern for poor penetration of the antimicrobials into the brain abscesses, linezolid was added for additional listeria coverage. After a 24-day hospital course, the patient was discharged to a long-term acute care hospital. The antibiotic regimen upon discharge consisted of ampicillin, gentamicin, and linezolid, with the plan to continue these medications for an additional four weeks, and the patient remained on ASMs.

## Discussion

Listeria cerebritis is a rare yet dangerous infection in immunocompromised patients. In these patients, the pathogen can lead to infection within the central nervous system and blood [2]. Infection is often clinically undetectable in patients with intact immune systems. It is often a result of ingesting meat and/or unpasteurized dairy products contaminated with the pathogen. The incidence of listeriosis, though uncommon, has increased in recent years, with a mortality rate of nearly 20% [1,7].

Pleocytosis and elevated protein can be seen in the CSF profile, but in many cases, such as the one described above, the CSF may be bland. CSF culture can grow listeria even if the CSF profile is only mildly abnormal.

MRI with contrast may show hyperintense lesions on T2-weighted images and enhancing lesions on T1-weighted images in the cerebral parenchyma [3]. Brain imaging can also show abscesses, brain edema, hydrocephalus, and ischemia.

Given the initially negative infectious workup and our patient’s history of cancer, our patient’s diagnosis was presumed to be leptomeningeal metastasis of melanoma, an often fatal condition. The initial CSF profile was only significant for mildly elevated protein, and the CSF culture showed no growth. Head imaging demonstrated nonspecific findings of subtle leptomeningeal/cortical enhancement and right parasagittal convexity. Given the high index of suspicion for malignancy and the patient’s rapidly deteriorating condition, a biopsy was pursued. Only after the biopsy did the patient become febrile. Tissue culture and pathology confirmed the diagnosis of listeria cerebritis, a condition with a promising response to antibiotics. The standard antibiotic regimen for treating listeria cerebritis combines intravenous ampicillin or penicillin plus gentamicin [1]. Individual patient characteristics such as immunocompetence play an important role in guiding the recommended duration of treatment [8]. Patients who are immunosuppressed may require up to three weeks of gentamicin therapy as well as anywhere between four to eight weeks of treatment with ampicillin or penicillin [4,8]. As with our patient, additional coverage with linezolid may also be considered. However, there remains limited evidence to support this [8].

## Conclusions

Listeria cerebritis is often confused with other conditions, including leptomeningeal carcinomatosis. Listeriosis is also more common in patients on immunosuppressive therapy. Therefore, biopsy may be helpful in distinguishing these two conditions if the CSF is otherwise bland. Patients and providers can utilize the biopsy results to make more educated care decisions. It is critical to have a high index of suspicion for listeria cerebritis in patients who are immunocompromised with focal imaging abnormalities, abscess formation, and/or focal seizures with fevers. This report describes one case of listeria cerebritis, and future literature should include additional descriptions of listeria cerebritis in cases without red flag signs for the diagnosis.

## References

[1] (2023). Listeria (Listeriosis): Information for Health Professionals and Laboratories. https://www.cdc.gov/listeria/technical.html#:~:text=Nearly%20everyone%20with%20listeriosis%20is,or%20death%20of%20the%20newborn.

[2] Gelfand MS, Swamy GK, Thompson JL (2023). Epidemiology and pathogenesis of Listeria monocytogenes infection. UpToDate.

[3] Gelfand MS, Swamy GK, and Thompson JL (2024). Clinical manifestations and diagnosis of Listeria monocytogenes infection. UpToDate.

[4] Charlier C, Perrodeau É, Leclercq A (2017). Clinical features and prognostic factors of listeriosis: the MONALISA national prospective cohort study. The Lancet.

[5] Lauko A, Rauf Y, Ahluwalia MS (2020). Medical management of brain metastases. Neurooncol Adv.

[6] Mauritz M, Hirsch LJ, Camfield P, Chin R, Nardone R, Lattanzi S, Trinka E (2022). Acute symptomatic seizures: an educational, evidence-based review. Epileptic Disord.

[7] Choi MH, Park YJ, Kim M (2018). Increasing incidence of listeriosis and infection-associated clinical outcomes. Ann Lab Med.

[8] Gelfand MS, Swamy GK, and Thompson JL (2024). Treatment and prevention of Listeria monocytogenes infection. UpToDate.

